# Insights into the enumeration of mixtures of probiotic bacteria by flow cytometry

**DOI:** 10.1186/s12866-023-02792-2

**Published:** 2023-02-27

**Authors:** Harry Tracey, Niall Coates, Eleri Hulme, Daniel John, Daryn Robert Michael, Susan Frances Plummer

**Affiliations:** Cultech Limited, Unit 2 Christchurch Road, Baglan Industrial Park, Port Talbot, UK

**Keywords:** Probiotic, Flow cytometry, Plate count, Multistrain

## Abstract

The use of flow cytometry to enumerate microorganisms is gaining traction over the traditional plate count technique on the basis of superior accuracy, precision and time-to-result. Here, we assessed the suitability of live/dead flow cytometry for the enumeration of mixed populations of probiotic bacteria (*L. acidophilus*, *L. paracasei, L. plantarum*, *L. salivarius, B. lactis* and *B. bifidum)* whilst comparing outcomes with plate counting. Using a novel gating strategy designed specifically for the enumeration of mixed populations, the application of flow cytometry resulted in the detection of higher numbers of viable bacteria with a greater level of repeatability than plate counting (RSD of 6.82 and 13.14% respectively). Across all multi-species blends tested, viable cell input was more accurately recovered by flow cytometry (101.8 ± 6.95%) than plate counts (81.37 ± 16.03%). However, when certain probiotic mixtures contained preparations with high numbers of non-viable cells in their total population, flow cytometry had the potential for overestimation of the viable population. Nevertheless, the comparative plate counts of these mixtures were low and variable, thus supporting the use of flow cytometry for the enumeration of viable bacteria in mixed populations.

**Supplementary Information:**

The online version contains supplementary material available at 10.1186/s12866-023-02792-2.

## Introduction

The enumeration of bacterial preparations using the plate count culture technique has been in place since the nineteenth century [1]. The method involves the cultivation of organisms on solidified growth medium and counting individual colonies to determine the number of Colony Forming Units (CFUs) as a representation of the viable cells present [2]. The technique is reliable but quite labour-intensive, with a measure of uncertainty ranging from 10 to 20% and can involve incubation periods of up to 5 days prior to enumeration. Culturing conditions can also pose problems – for example anaerobic organisms that require stringent handling conditions. Alternative enumeration techniques have been explored over the years [3, 4] but none has been widely adopted.

The interest in probiotic nutritional supplements, comprising ‘live micro-organisms which, when administered in adequate amounts, confer a health benefit on the host’ [5], along with the growth of the probiotic market sector has drawn attention to bacterial enumeration techniques. Non-culture based enumeration methods such as live/dead flow cytometry (FC) have gained much traction due to the potential for rapid enumeration, a superior analytical precision (repeatability and reproducibility) and the ability to quantify the viable but non-culturable (VBNC) bacterial population [3, 6, 7]. The ISO19344|IDF 232(2015) method [8] for the quantification of lactic acid bacteria in starter cultures, probiotics and fermented products involves dual nucleic acid staining with two coloured fluorescent dyes, of which one is cell permeant, the other not. The permeant dye penetrates the membranes of all cells to stain the intracellular nucleic acids whereas the non-permeant dye enters only those cells with damaged membranes (non-viable) where it displaces the permeant dye due to a higher affinity for nucleic acid binding. The flow cytometer quantifies the degree of light scattering and emitted fluorescence for each cell, allowing determination of morphological and fluorescent properties. Viable bacteria (Active Fluorescent Units (AFUs)) with intact membranes, fluoresce bright green whereas non-viable organisms (non-Active Fluorescent Units, (n-AFUs)) are indicated by red fluorescence.

The data generated by the flow cytometer must be segregated using ‘gates’ to separate cells from the background noise and distinguish the viable populations from non-viable. However, the positioning of these gates is subjective and organism specific – determined by morphological characteristics such as size, shape and granularity [3] as well as genome size and GC content [9]. A number of studies have demonstrated that FC can be used effectively to enumerate viable bacteria in pure cultures [6, 7, 10, 11] but there has been less consideration of applying the same principles to enumerate viable bacteria in mixed populations [12]. There is growing consumer pressure for probiotic manufacturers to formulate products containing a plethora of different organisms but the question is - can these be enumerated effectively using live/dead flow cytometry? Here we use plate counting and flow cytometry to enumerate lactic acid bacteria and bifidobacteria, comparing the efficacy and precision of the different techniques. We then propose the use of a general gating system that could be applied for the enumeration of mixtures of organisms, and compare its application with results from the plate count method in order to assess the usefulness of flow cytometry for the enumeration of viable bacteria in multi-species probiotic blends.

## Methods

### Reagents and materials

Reagents were purchased from Sigma-Aldrich (Dorset, UK) unless otherwise stated. Bacterial culture media was purchased from Oxoid Ltd. (Basingstoke, UK) unless otherwise stated.

### Probiotic preparations

The probiotic organisms analysed in this study were co-cultures *Lactobacillus acidophilus* (NCIMB 30156) and *Lactobacillus acidophilus* (NCIMB 30157), referred to as CUL21/60 and *Bifidobacterium bifidum* (NCIMB 30153) and *Bifidobacterium animalis subsp. lactis* (NCIMB 30172), referred to as CUL20/34 as well as pure cultures of *Lacticaseibacillus paracasei* CUL08 (NCIMB 30154), *Lactiplantibacillus plantarum* CUL66N (NCIMB 30280) and *Ligilactobacillus* s*alivarius* CUL61 (NCIMB 30211). All organisms were provided as freeze dried powders by Cultech Ltd. for analysis.

### Enumeration of viable bacteria by plate count (PC) testing

Viable bacterial numbers were assessed using a modified version of the Miles and Misra plate count technique [2]. A 10^− 1^ dilution of the organism in Maximum Recovery Diluent (MRD) was mixed for 15 minutes on a roller mixer at room temperature. A decimal dilution series was prepared in MRD and 10 × 10 μl of an appropriate dilution was plated on DeMan Rogosa Sharpe (MRS) agar for lactobacilli or MRS-X (MRS containing lithium chloride (1 g/L), sodium propionate (1.5 g/L) and L-cysteine hydrochloride (0.25 g/L)) for bifidobacteria. Inoculated plates were incubated anaerobically (10% carbon dioxide, 5% hydrogen and 85% nitrogen) at 37 °C for 72 hours. Results are expressed as the number of colony forming units (CFU) per gram of sample.

### Enumeration of viable and non-viable bacteria by flow cytometry

The numbers of viable and non-viable cells were determined using a BD Accuri™ C6 Plus flow cytometer (BD BioSciences, New Jersey, USA) in accordance with Protocol B of ISO 19344|IDF 232(2015) [8] but with a slight modification; the dilution series were prepared in MRD as per the plate count method (rather than using Peptone). The flow cytometer was calibrated before every session with BD CS&T quality control beads (BD BioSciences, New Jersey, USA). The fluorescent dyes, Propidium Iodide (PI) (non-permeant, red dye) and SYTO™24 (permeant, green dye) were purchased from Invitrogen (Massachusetts, USA) and diluted in filtered DI water to generate working stocks of 0.2 mM and 0.1 mM respectively. Freeze-dried probiotic preparations were diluted to 1–5 × 10^7^ total fluorescent units (tAFU)/ml and 100 μl of this preparation was added to a solution of 880 μl MRD and 10 μl of both fluorescent dyes followed by a 15 minute incubation in the dark at 37 °C. Samples were vortexed immediately before analysis on the flow cytometer with the following settings: 50 μl uptake, ‘medium’ fluidics (35 μl min^− 1^ flow rate achieving 1000–2000 events per second), excitation by the 488 nm blue laser and only data exceeding a primary threshold of 2500 FSC-H and secondary threshold of 1000 Syto24-H were collected. Data was analysed with multi-parametric dot plots using the BD Accuri C6 Plus software (BD BioSciences, New Jersey, USA). Doublets were identified on a forward scatter area vs forward scatter height (FSC detector) cytogram and retained in the analysis after being found to represent only a negligible fraction of events (< 1% - Supplementary fig. S1). Bacterial events were separated from background noise using a forward scatter vs side scatter (SSC detector) cytogram and viability was assessed with green (FL-1 detector)/red (FL-3 detector) fluorescence. Green fluorescent cells were considered viable (Active Fluorescent Units, AFU) whereas the red fluorescent cells and any double-stained (green and red) cells were considered to be non-viable (non-Active Fluorescent Units, n-AFU). Data are expressed as AFU or n-AFU per gram of sample. FCS files representative of the flow cytometry dataset have been made publically available at FlowRepository [13] (https://flowrepository.org/id/FR-FCM-Z632).

### Enumeration of viable but non-culturable bacteria (VBNC)

To obtain the numbers representing the VBNC population, the numbers obtained from the PC (CFU/g) were subtracted from the viable numbers obtained from FC (AFU/g) for each preparation.

### Precision (repeatability) analysis

The relative standard deviation (RSD) of data sets was calculated by dividing the standard deviation of experimental replicates by the mean of the experimental replicates and then multiplying by 100, expressing results as a percentage. Assays with RSD < 15% are considered to be precise [7].

### Formulation of multistrain probiotic blends

Freeze-dried preparations were mixed in a variety of combinations to create the multi-species blends (Table 1) with the aim of achieving a total of 4 × 10^10^ AFU/g for each blend. Constituent preparations were enumerated by FC and PC before mixing, and the average AFU/g of a minimum of 3 weighing repeats was used to generate the blends of 4 × 10^10^ AFU/g. Maltodextrin (MD20) was used as the excipient for the preparation of the blends.

**Table 1:** Composition of the multi-species blends analysed in this study

Blend	Composition	% AFU input
**1**	CUL20/34	20
	CUL21/60	80
**2**	CUL20/34	50
	CUL61	50
**3**	CUL20/34	25
	CUL21/60	25
	CUL08	25
	CUL66N	25
**4**	CUL20/34	20
	CUL21/60	20
	CUL61	20
	CUL08	20
	CUL66N	20

*AFU* Active fluorescence units

### Statistical analysis

The normality of the data sets was confirmed using the Shapiro-Wilks test and/or visual inspection of Q-Q plots and statistical differences determined using the two-tailed paired Student’s t-test. All statistics were performed using GraphPad PRISM (Version 9.0.2, California, USA) and values of *p* less than 0.05 were considered to be statistically significant.

## Results

**Fig. 1:**
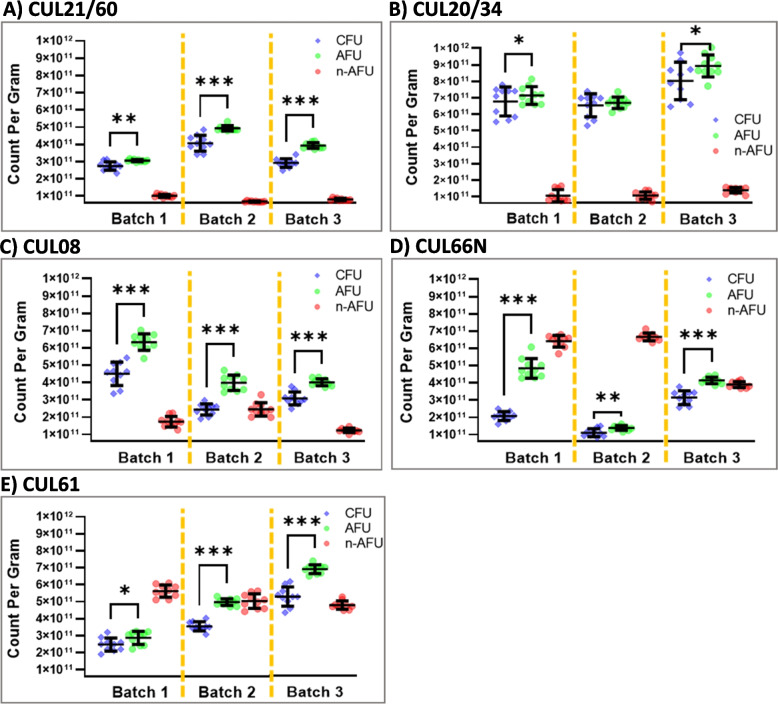
Quantification of bacterial numbers present in probiotic preparations. Bacterial numbers present in 3 batches of **A** CUL21/60, **B** CUL20/34, **C** CUL08, **D** CUL66N or **E** CUL61 were determined by PC (CFU/g) and FC (AFU/g and n-AFU/g). Data represent the mean ± SD of 10 experimental replicates per batch. Values of *p* were determined using the Student’s paired t-test where *p < 0.05, **p < 0.01 and ***p < 0.001. *Abbreviations:* PC, plate count; FC, flow cytometry; CFU, colony forming unit; AFU, active fluorescent unit; n-AFU non-active active fluorescent unit; SD, standard deviation

Figure 1 illustrates the viable numbers present in each bacterial preparation when enumerated using the Plate Count (PC) technique and the numbers of viable and non-viable cells using the Flow Cytometry (FC) technique*.* Numbers of CFU/g (PC), AFU/g (FC) and n-AFU/g (FC) varied depending on the organism but the viable numbers generated by FC and PC showed that AFU/g were consistently significantly higher than CFU/g. CUL20/34 (Fig. 1B) contained the highest number of viable cells (both CFU/g and AFU/g) whilst CUL66N (Fig. 1D) and CUL61 (Fig. 1E) contained the highest numbers of non-active cells (n-AFU) but batch-to-batch variation was observed for all preparations. The proportion of VBNC bacteria detected varied from batch-to-batch but appeared to be organism specific.

The precision (repeatability) of the FC and PC techniques is shown in Table 2 with RSD values ranging from 3.14 to 8.67% for FC and 9.66 to 15.41% for PC. The overall RSD for FC was 6.70% - nearly half of the 12.52% observed for PC (*p* = 0.0002, Table 2). The accuracy of all assays was assessed by linear regression analysis and the R^2^ values exceeded 0.9 (Supplementary Fig. S2).

**Table 2:** Precision analysis of PC and FC

	RSD (%)		***p*** value
	FC	PC	
**CUL21/60**	3.14	9.66	0.0276
**CUL20/34**	6.74	12.62	0.0043
**CUL08**	7.87	13.55	0.0872
**CUL66N**	8.67	15.41	0.1672
**CUL61**	7.08	11.36	0.0997
**Mean (SD)**	6.70(2.13)	12.52(2.18)	0.0002

Data represents the mean of 3 batches (10 experimental replicates per batch). Values of *p* were determined using the Student’s paired t-test

*Abbreviations: RSD* Relative standard deviation, *FC* Flow cytometry, *PC* Plate count, *SD* Standard deviation

**Fig. 2:**
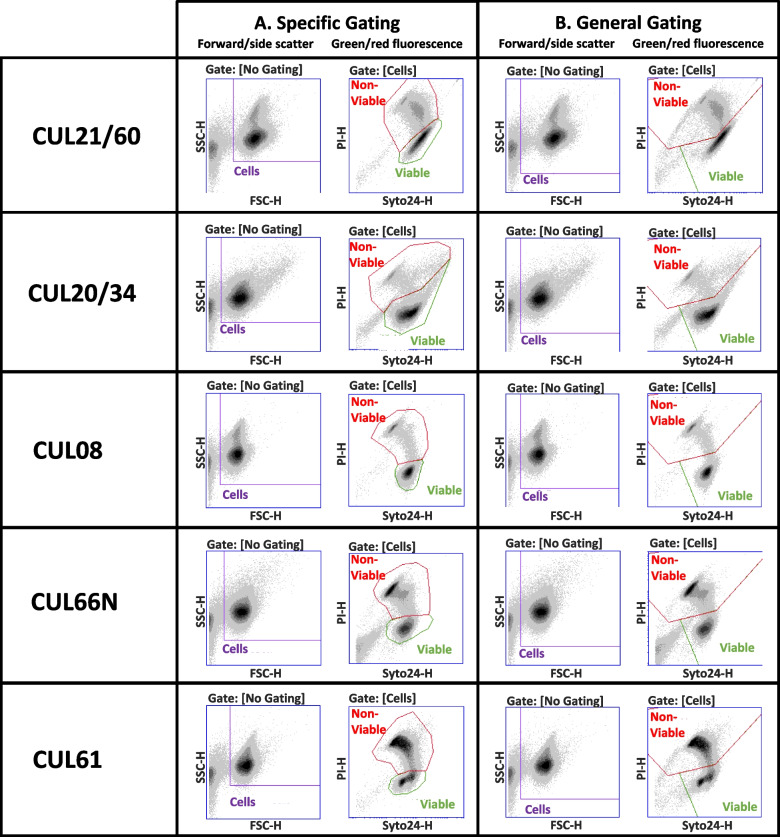
Specific and general flow cytometric gating strategies. Representative flow cytometric multi-parameter dot plots (forward (FSC-H)/side (SSC-H) scatter and green (SYTO24-H)/red (PI-H) fluorescence) for each organism overlaid with **A** the specific gates or **B** a general gating strategy

The forward/side scatter profiles seen in Fig. 2A (and Supplementary Fig. S4A) illustrate that each organism has a unique pattern that requires its own specific gating to optimise separation of the cells from any background “noise”. Similarly, the green/red fluorescence plots indicate organism specific profiles (Fig. 2A and Supplementary Fig. S4B). A more comprehensive presentation of the specificity is seen in Supplementary Fig. S4 which details each organism against every other organism’s specific gating. The specific gating strategies were edited to generate a single ‘general’ gating strategy with the aim of enumerating any of the organisms without compromising accuracy (shown in Fig. 2B). Comparisons of enumeration achieved using the general gates with the results obtained with the specific gates resulted in differences of no more than 2% for CUL20/61, CUL20/34 and CUL08; < 5% for CUL66N and < 11.5% for CUL61 (Table 3). CUL61 (*L. salivarius*) carries a “tail” of double stained non-viable bacteria that appears to fall within both the non-active and active sectors of the general gating strategy which is expressed as an “overestimation” of AFU.

**Table 3:** Comparison of specific and general gating strategies for the enumeration of viable bacteria

	AFU per gram		% Difference
	Specific gating	General gating	
CUL21/60			
Batch 1	3.06 × 10^11^	3.06 × 10^11^	− 0.06
Batch 2	3.95 × 10^11^	3.98 × 10^11^	+ 0.67
Batch 3	4.96 × 10^11^	4.92 × 10^11^	−0.79
Mean (SD)			−0.06(0.70)
CUL20/34			
Batch 1	7.14 × 10^11^	7.10 × 10^11^	−0.62
Batch 2	6.43 × 10^11^	6.32 × 10^11^	−1.72
Batch 3	8.57 × 10^11^	8.52 × 10^11^	−0.56
Mean (SD)			−0.97(0.65)
CUL08			
Batch 1	6.33 × 10^11^	6.41 × 10^11^	+ 1.27
Batch 2	3.98 × 10^11^	4.08 × 10^11^	+ 2.47
Batch 3	4.01 × 10^11^	4.06 × 10^11^	+ 1.44
Mean (SD)			+ 1.73(0.65)
CUL66N			
Batch 1	4.84 × 10^11^	5.02 × 10^11^	+ 3.64
Batch 2	1.40 × 10^11^	1.47 × 10^11^	+ 4.90
Batch 3	4.14 × 10^11^	4.35 × 10^11^	+ 4.87
Mean (SD)			+ 4.47(0.72)
CUL61			
Batch 1	2.86 × 10^11^	3.33 × 10^11^	+ 14.01
Batch 2	4.98 × 10^11^	5.49 × 10^11^	+ 9.42
Batch 3	6.90 × 10^11^	7.67 × 10^11^	+ 10.04
Mean (SD)			+ 11.16(2.50)

Data represents the mean of 10 experimental replicates per batch

*Abbreviations: AFU* Active fluorescence units, *SD* Standard deviation

**Fig. 3:**
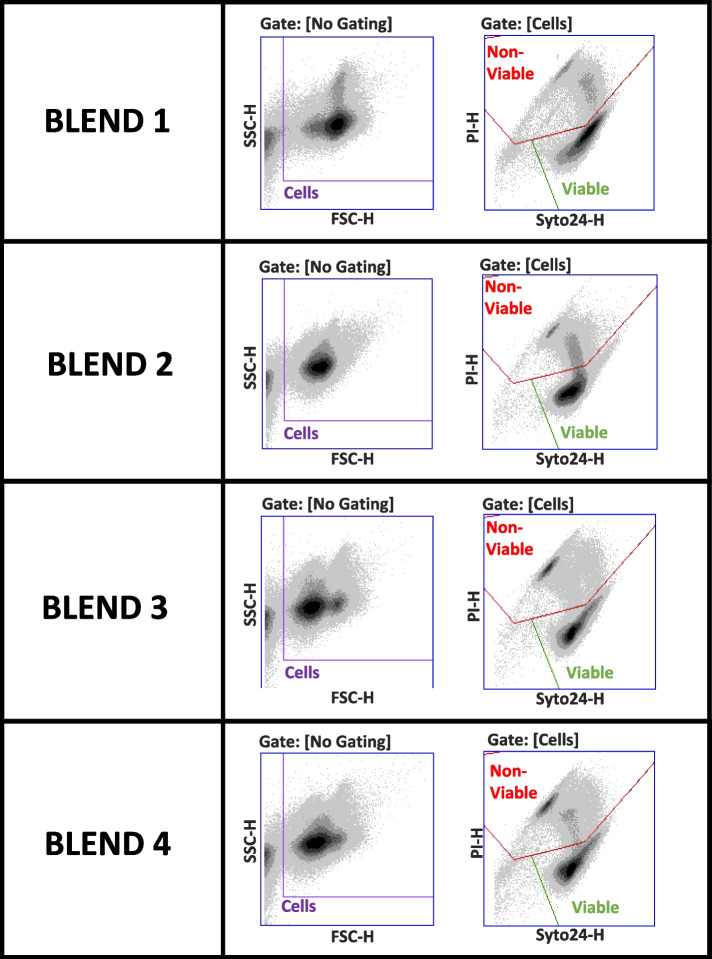
General gating strategy for probiotic blends. Representative flow cytometric multi-parameter dot plots (forward (FSC-H)/side (SSC-H) scatter and green (SYTO24-H)/red (PI-H) fluorescence) for each of the blends overlaid with the general gating strategy

When applying the general gating strategy to enable enumeration of mixed populations, the blend comprising 80% *Lactobacillus acidophilus* and 20% bifidobacteria (Blend 1, Table 4) achieved comparable recoveries using both techniques. Substitution of *L. acidophilus* with *L. salivarius* altered the recovery substantially with only 58% of the expected numbers recovered using PC but > 100% recovered by FC (Blend 2). The introduction of more complexity to the mixes (Blends 3 & 4) indicated that the generalized gating supported nearly 100% recovery versus expected using FC but that the PC method was less successful at supporting the expected recovery of the total microbial population, particularly when *L. salivarius* was included in the formulation (Blend 4, Table 4). Figure 3 details the FC plots for the 4 blends showing that the forward/side scatter plots appear to capture the population effectively but the green/red fluorescence plots are less clear. Blend 2 illustrates the “tail” of double stained non-viable *L. salivarius* that is believed to contribute to the “over-recovery” observed for the FC with this blend, but the extent of the “tail” ingression cannot be determined. *L. salivarius* is also included in Blend 4 (at lower proportion) and there are indications of a similar recovery pattern with this blend.

**Table 4:** Quantification of bacterial numbers present in multi-species probiotic blends

	FC				PC				***p***-values (FC vs PC)	
	Formulated AFU/g	Recovered AFU/g	RSD (%)	% Recovery	Formulated CFU/g	Recovered CFU/g	RSD (%)	% Recovery	Recovered counts/g	% Recovery
**Blend 1**	3.85 × 10^10^	3.66 × 10^10^	9.62	95.22	3.00 × 10^10^	2.78 × 10^10^	10.86	92.75	0.0054	< 0.0001
**Blend 2**	3.62 × 10^10^	4.04 × 10^10^	7.34	111.56	3.02 × 10^10^	1.76 × 10^10^	14.60	58.18	0.0001	0.0003
**Blend 3**	4.33 × 10^10^	4.30 × 10^10^	6.86	99.43	3.39 × 10^10^	3.10 × 10^10^	15.62	91.40	< 0.0001	0.0088
**Blend 4**	4.30 × 10^10^	4.35 × 10^10^	3.44	101.08	3.33 × 10^10^	2.76 × 10^10^	11.47	83.13	< 0.0001	0.0010
**Mean (SD)**			6.82 (2.55)	101.8 (6.945)			13.14 (2.33)	81.37 (16.03)		

Data represents the mean of at least 5 experimental repeats. Values of *p* were determined using the Student’s paired t-test

*Abbreviations: AFU* Active fluorescence units, *CFU* Colony forming units, *RSD* Relative standard deviation, *FC* Flow cytometry, *PC* Plate count, *SD* Standard deviation

## Discussion

This study compared the enumeration of lactic acid bacteria and bifidobacteria as pure and blended cultures using live/dead flow cytometry and plate counting. FC was found to have superior precision and recovery capabilities compared to PC; we assessed a number of freeze-dried probiotic preparations using both methods and showed that FC achieved consistently significantly higher viable counts than PC - indicating the presence of VBNC bacteria which have been noted previously [10, 14] had superior precision (6% vs 12% respectively) thus adding to a growing body of evidence demonstrating the benefits of FC for probiotic enumeration [3, 6, 7]. In our mixed populations containing variable numbers of viable and non-viable cells it appeared that overestimations of the viable numbers could occur with the FC method but did not occur with the PC technique. For FC the recovery against expected input for all blends was within the range of 95–112% of input whilst the PC recoveries ranged from 58 to 93% of expected. It seems that when CUL61 was included in a multi-species formulation an overestimation of the viable numbers occurred with FC but the result using PC was much lower than expected. In line with the enumeration of the pure cultures, for the blends, FC displayed less variability than PC, with average RSD values across all blends of 7 and 12% respectively.

FC, unlike PC, has the ability to detect populations of non-viable cells and we identified considerable variations; low proportions of non-viable cells for CUL21/60, CUL20/34 and CUL08 but higher proportions for CUL66N and CUL61. Similar proportions and variation in non-viable cells were observed by Lugli et al using live/dead FC to enumerate commercially available probiotics products [12]. There are two populations included in the “non-viable” category - one stained only with PI, the other double stained with both Syto24 and PI – and these populations sit adjacent to the viable (Syto24 only) population on a fluorescent light plot. To achieve an accurate estimation of AFU, the viable gate separates the viable and double-stained populations by marking a narrow border between the two. Marginal increases in PI fluorescence push a cell from the “viable” into the “non-viable” population. Fluorescence profiles vary between species, owing to aptitude for dye uptake, genome size and GC content [9], hence, each species has a unique optimal gating strategy. It is therefore feasible that the position of the viable population of one species can overlap with the non-viable population of another species - as is the case for CUL20/34 and CUL61 in this study.

Numerous studies have used FC to enumerate freeze-dried individual probiotic strains [3, 6, 15, 16] but there is a growing consumer demand for multi-strain probiotics and hence the need to explore the potential to identify/propose a general (“fit-for-all”) gating strategy. Owing to the individual nature of each species’ fluorescence profile, the shape of the general gate was dictated by the strains included in the mixed populations. Most of the preparations investigated in this study are similar enough that the general gates are akin to the specific gates, causing < 5% difference between the two strategies. However, despite sharing the same genus as most other preparations, CUL61 has a distinctive fluorescence profile, which, in combination with large proportions of double-stained cells, resulted in a ~ 11% overestimation of recovery. These findings highlight the need to consider each species individually prior to enumerating a multistrain population with live/dead FC; ad hoc creation of gates to fit a mixed population without prior consideration of their fit on the constituent species doesn’t allow for recognition of the gating strategy’s inaccuracies.

Future enumeration of single and mixed bacterial populations may be achieved with automated gating software. So far, the burgeoning field of computational flow cytometry has focussed on algorithms to streamline immuno-phenotyping [17], but there is increasing development in the microbial sector, primarily for identification of distinct phenotypes in ecologically complex microbial samples [18]. We are unaware of any pipelines designed for automated gating of dual fluorescent stained microbial populations which might be applied to live/dead enumeration. Although, the batch on batch consistency of routine probiotic enumeration as shown here, allows for the repeated application of a single gating strategy to multiple samples, making automation unnecessary; particularly if the algorithm requires training on user defined gates in the first place [18]. Should automated live/dead gating for bacteria reach an appropriate standard, future work will be conducted with this dataset to compare our gating strategies to an automated analysis.

This study is limited by the use of only one of three variations of the FC methods approved in ISO19344|IDF 232(2015) [8]; another technique may result in differing success of multi-species enumeration by live/dead flow cytometry. Additionally, the previously published [14] threshold and gating method we use to isolate cells from background noise, based on FSC-H excludes a population of events that have a similar granularity (SSC-H) to the events we consider to be bacterial cells. Future work is required to determine if this castigated minority population are actually small bacterial cells and determine whether we and others have slightly underestimated the number of bacteria in freeze-dried probiotic preparations. The general gating strategy we have proposed is specific to the organisms tested in this study and there is a need to expand this work to consider other organisms and mixtures to determine if this proposal is feasible on a broad scale. The use of species-specific antibodies may provide a more accurate means of enumerating mixtures of probiotic bacteria and represents a promising avenue of future work [19]. However, development of species-specific antibodies requires access to animal facilities with expert immunologists, are finite in abundance and may suffer from issues of cross-reactivity – all of which are barriers for acceptance as a routine enumeration technique in the probiotic industry.

In conclusion, the comparison of live/dead flow cytometry with plate counts for the enumeration of probiotic bacteria indicates differences (mostly increased numbers) favouring the FC technique. The apparent overestimation of the numbers of viable bacteria in certain multi-species blends suggests that the application of this technique requires consideration if it is to be widely adopted but it has clear benefits over the industry standard PC technique in terms of repeatability and recovery. The application of a fixed general gating strategy rather than ad hoc gating of multi-species products would remove a level of subjectivity and analyst variation which is desirable for quality assurance purposes. The findings from this work highlight the need for further studies with the enumeration of complex and diverse probiotic products by flow cytometry with a comparison to the Plate Count technique.

## Supplementary Information

**Additional file 1: Supplementary Fig. S1.** Doublet discrimination. Supplementary Fig. S2. Accuracy of FC and PC methods. **Supplementary Fig. S3.** Multi-parameter dot plots displaying specific gating strategies overlaid onto freeze-dried preparations that had been A. incubated with 70% ethanol for 15 minutes and B. untreated – nominally “0% dead” because no extra ethanol-killed cells were added. **Supplementary Fig. S4.** Specificity of specific gating strategy. **Supplementary Fig. S5.** Dot plots of background noise in bacteria-free media - cryoprotectants (trehalose, sucrose, NaCl, K2HPO4 and KH2PO4) suspended in maximum recovery diluent. **Supplementary Fig. S6.** Ungated multi-parameter dot plots of one sample of each freeze-dried preparation looking at scatter signals vs individual viability stains.

## Data Availability

All data generated or analysed during this study are included in this published article (and its supplementary information files).
