# Supplementary material for: Insights into the enumeration of mixtures of probiotic bacteria by flow cytometry
Source: BMC Microbiol. 2023 Feb 27;23:48. doi: 10.1186/s12866-023-02792-2 (PMC9969615; doi:10.1186/s12866-023-02792-2)
Supplement: Supplementary file 1 — Additional file 1: Supplementary Fig. S1. Doublet discrimination. Supplementary Fig. S2. Accuracy of FC and PC methods. Supplementary Fig. S3. Multi-parameter dot plots displaying specific gating strategies overlaid onto freeze-dried preparations that had been A. incubated with 70% ethanol for 15 minutes and B. untreated – nominally “0% dead” because no extra ethanol-killed cells were added. Supplementary Fig. S4. Specificity of specific gating strategy. Supplementary Fig. S5. Dot plots of background noise in bacteria-free media - cryoprotectants (trehalose, sucrose, NaCl, K2HPO4 and KH2PO4) suspended in maximum recovery diluent. Supplementary Fig. S6. Ungated multi-parameter dot plots of one sample of each freeze-dried preparation looking at scatter signals vs individual viability stains. [file 12866_2023_2792_MOESM1_ESM.docx]

# *SUPPLEMENTARY INFORMATION*

**Insights into the enumeration of mixtures of probiotic bacteria by flow cytometry**

Harry Tracey, Niall Coates, Eleri Hulme, Daniel John, Daryn Robert Michael and Susan Frances Plummer


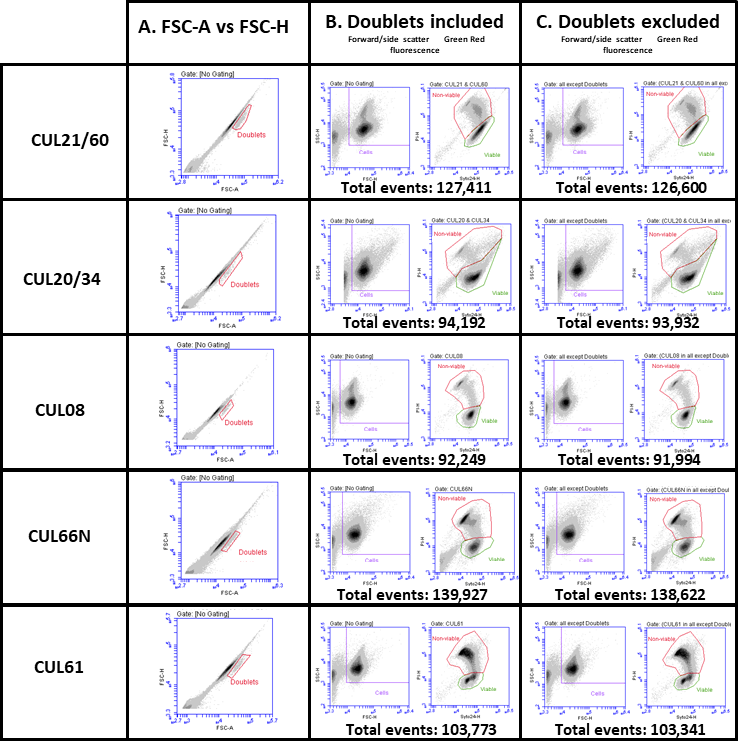


**Supplementary Figure S1. Doublet discrimination.** Forward Scatter Area (FSC-A) vs Forward Scatter Height (FSC-H) doublet analysis plots (A) and multi-parameter dot plots showing the enumeration of (B) freeze-dried probiotic preparation with doublets and (C) freeze-dried probiotic preparation without doublets.


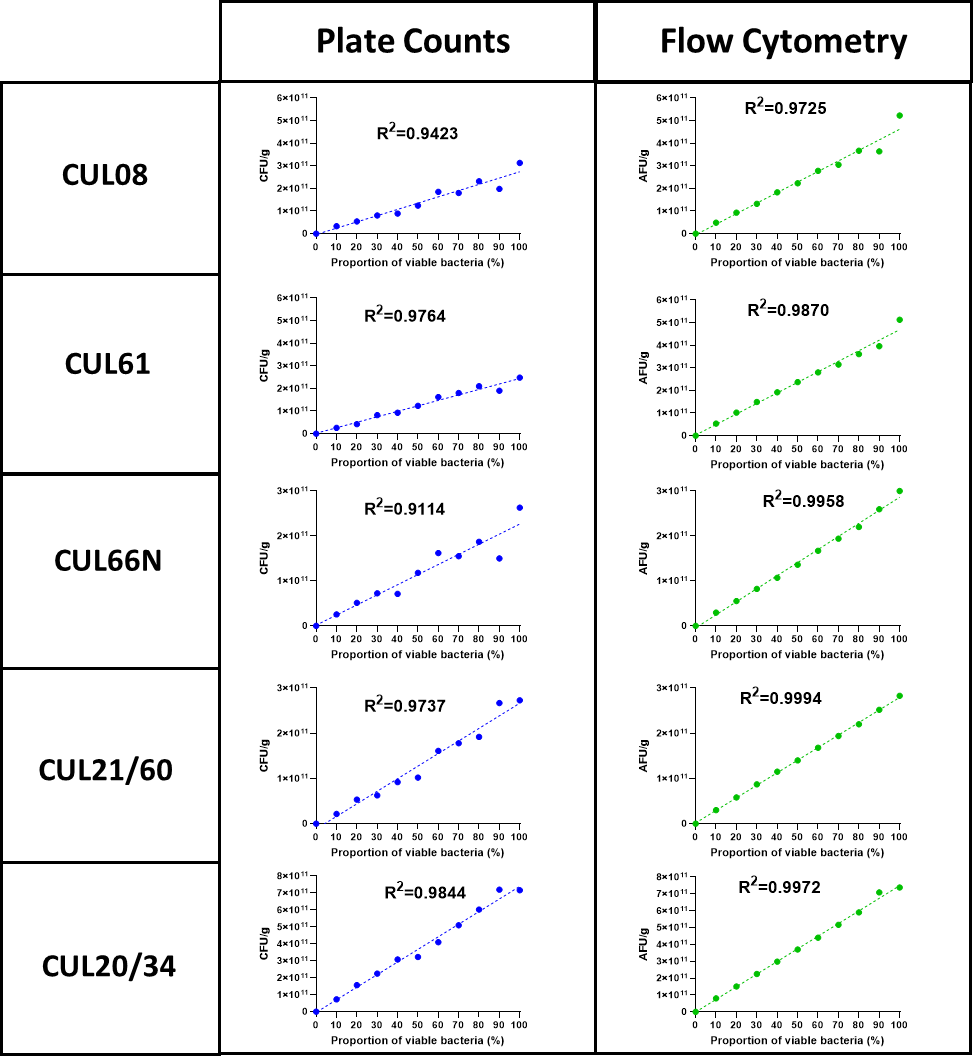


**Supplementary Figure S2. Accuracy of FC and PC methods.** Viable bacteria were mixed with ethanol killed bacteria (exposed to 70% ethanol for 15 minutes at room temperature) in proportions ranging between 0 and 100%. The numbers of viable bacteria present within each mixture were quantified by plate count and flow cytometry and the data analysed by linear regression with R^2^ values as stated. The data represents the mean of duplicate samples from a single experiment. *Abbreviations:* CFU, colony forming units; AFU, active fluorescent units.


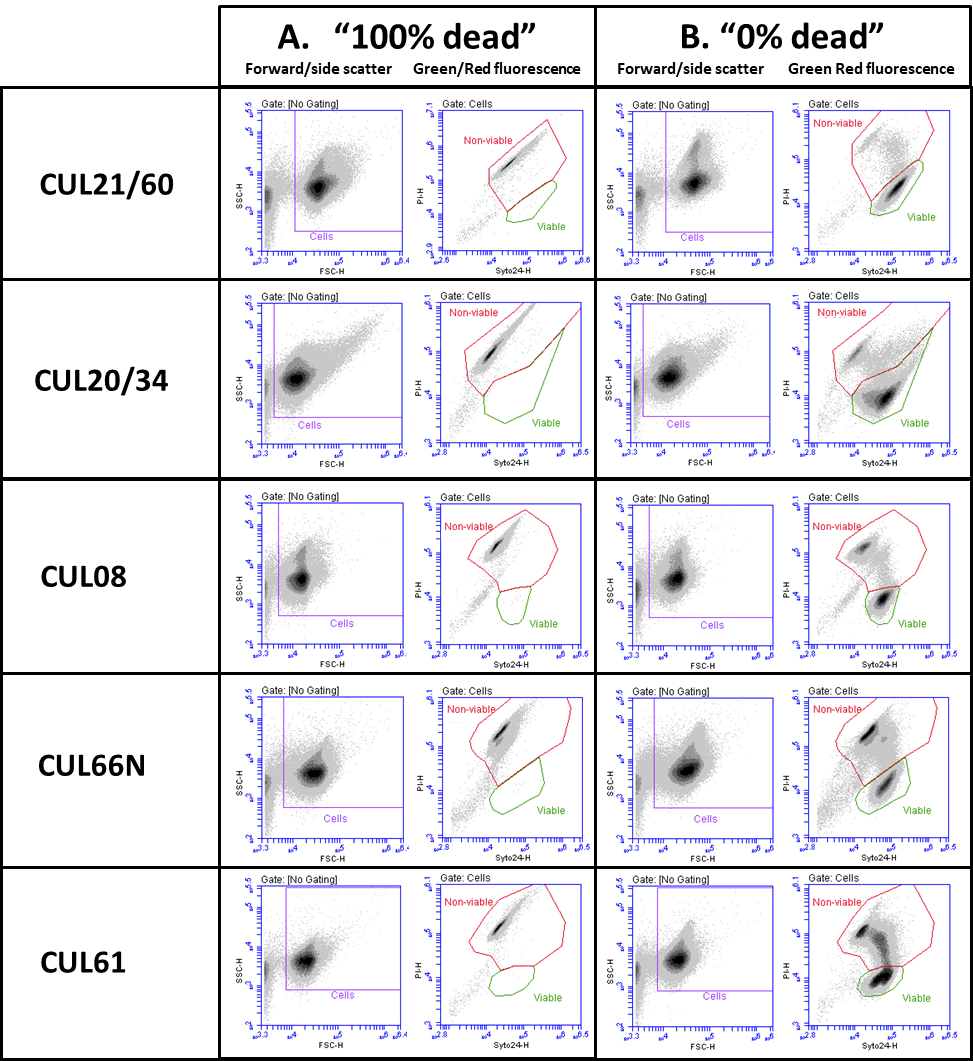


**Supplementary Figure S3.** Multi-parameter dot plots displaying specific gating strategies overlaid onto freeze-dried preparations that had been A. incubated with 70% ethanol for 15 minutes and B. untreated – nominally “0% dead” because no extra ethanol-killed cells were added.

**
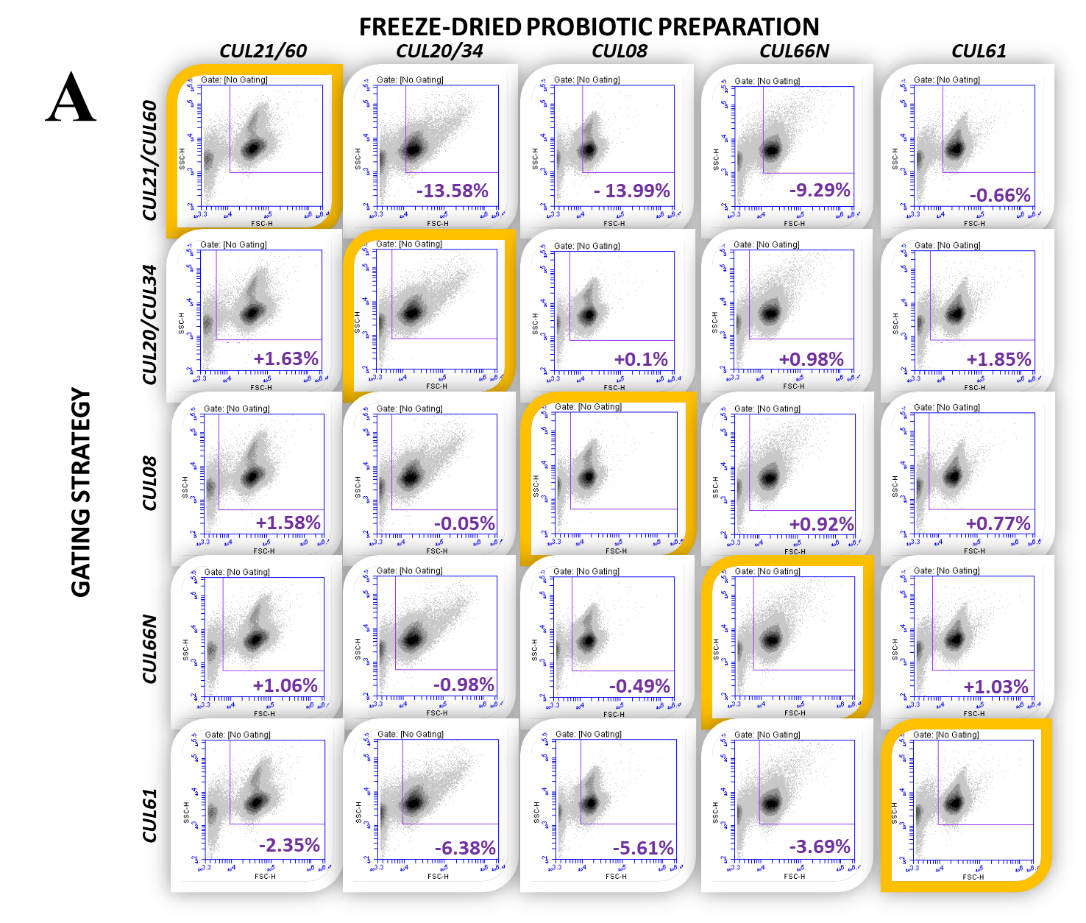
**


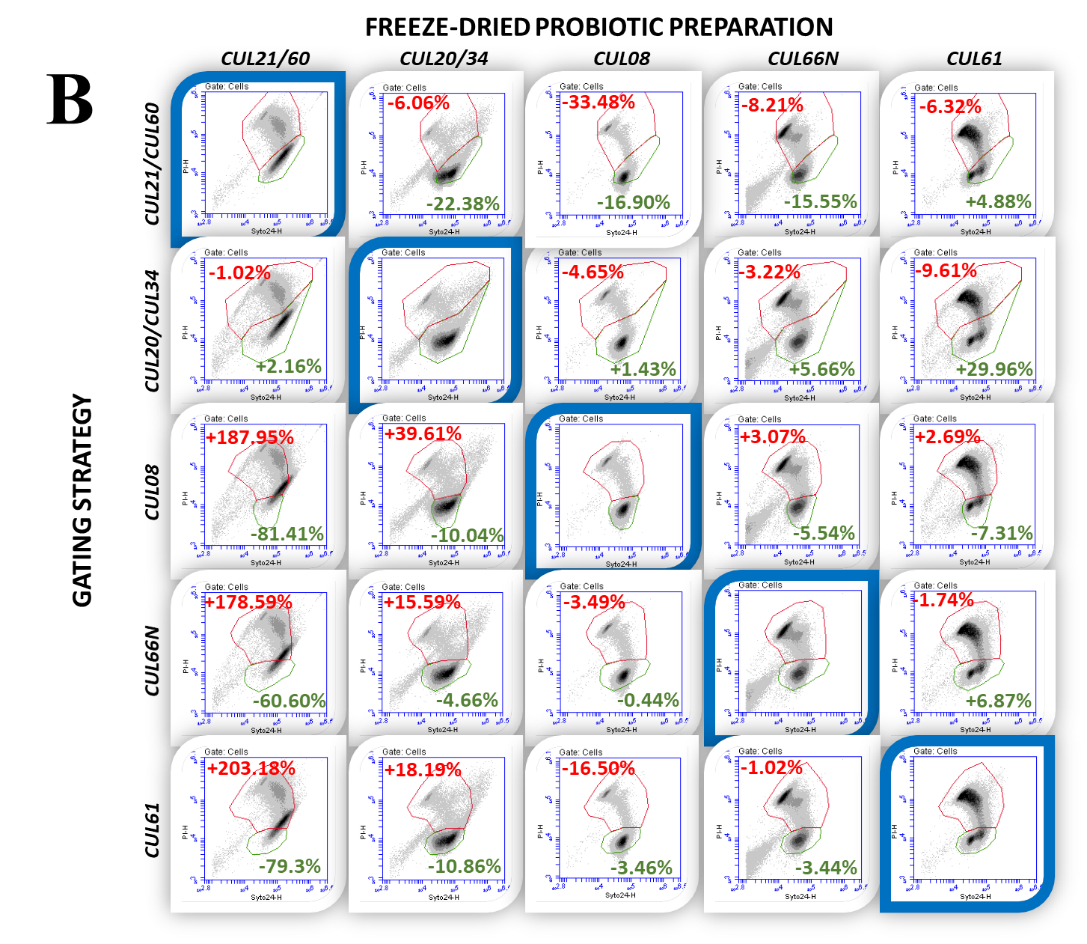


**Supplementary Figure S4. Specificity of specific gating strategy.** Matrices of flow cytometric multi-parameter dot plots of each freeze-dried probiotic preparation (FDPP) overlaid with the FDPP specific gating strategies. FDPP overlaid with the appropriate gating strategy are highlighted. (A) forward/side scatter and (B) green/red fluorescence gated with equivalent forward/side scatter gate in (A). Values represent enumeration of: cells (A), AFU and n-AFU (B) compared to the appropriate gating.


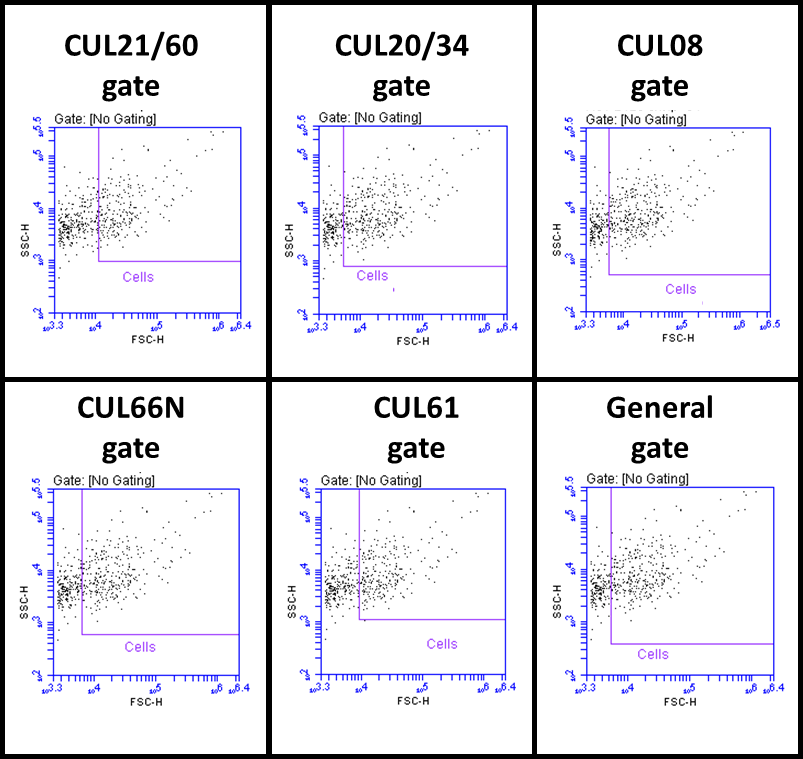


**Supplementary Figure S5.** Dot plots of background noise in bacteria-free media - cryoprotectants (trehalose, sucrose, NaCl, K2HPO4 and KH2PO4) suspended in maximum recovery diluent.

**CUL21/60**

**CUL20/34**

**CUL08**

**CUL66N**

**CUL61**


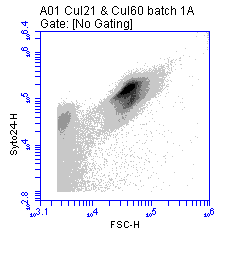


**A. FSC vs Syto**

**B. FSC vs PI**

**C. SSC vs Syto**

**D. SSC vs PI**


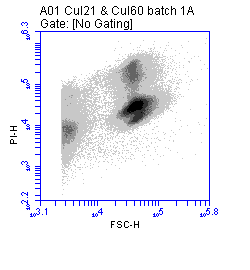

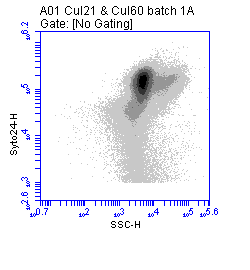

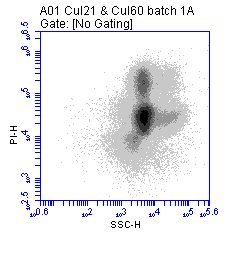

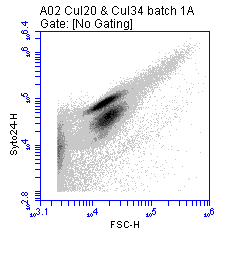

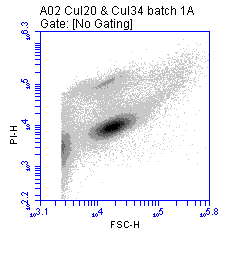

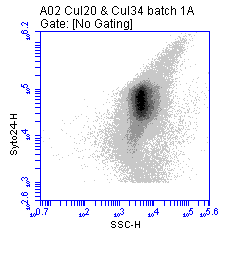

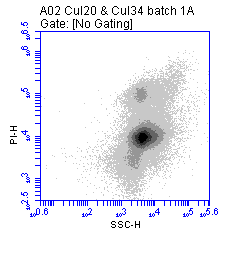

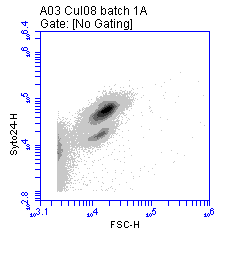

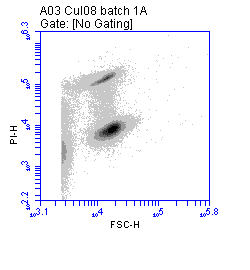

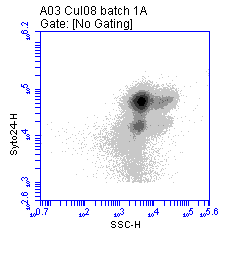

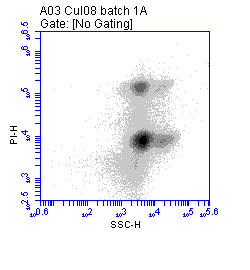

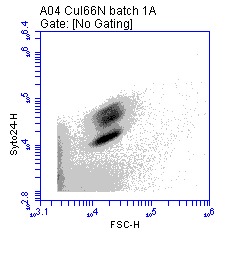

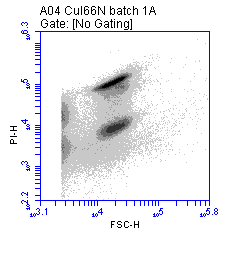

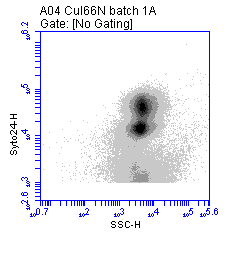

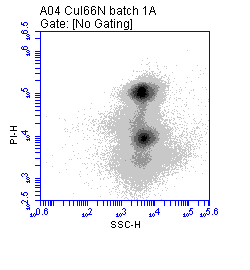

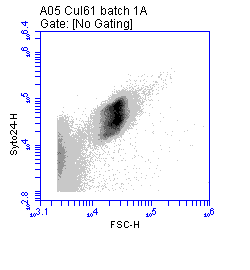

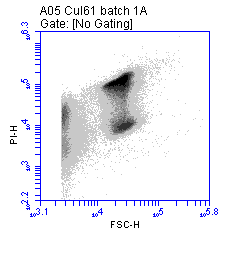

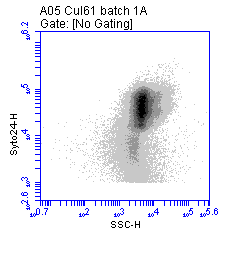

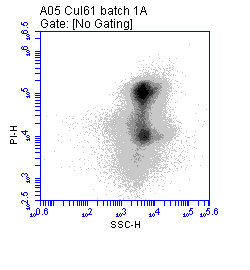


**Supplementary Figure S6.** Ungated multi-parameter dot plots of one sample of each freeze-dried preparation looking at scatter signals vs individual viability stains.
